# A developmental trajectory supporting the evaluation and achievement of competencies: Articulating the Mastery Rubric for the nurse practitioner (MR-NP) program curriculum

**DOI:** 10.1371/journal.pone.0224593

**Published:** 2019-11-07

**Authors:** Rochelle E. Tractenberg, Melody R. Wilkinson, Amy W. Bull, Tiffany P. Pellathy, Joan B. Riley

**Affiliations:** 1 Collaborative for Research on Outcomes and –Metrics, and Departments of Neurology, Biostatistics, Bioinformatics & Biomathematics, and Rehabilitation Medicine, Georgetown University, Washington, D.C., United States of America; 2 Department of Advanced Nursing Practice, School of Nursing & Health Studies, Georgetown University, Washington, D.C., United States of America; 3 Department of Nursing and Human Science, School of Nursing & Health Studies, and Center for New Designs in Learning and Scholarship, Georgetown University, Washington, D.C., United States of America; University of South Australia, AUSTRALIA

## Abstract

**Background:**

Advanced practice registered nursing (APRN) competencies exist, but there is no structure supporting the operationalization of the competencies by APRN educators. The development of a Mastery Rubric (MR) for APRNs provides a developmental trajectory that supports educational institutions, educators, students, and APRNs. A MR describes the explicit knowledge, skills, and abilities as performed by the individual moving from novice (student) through graduation and into the APRN career.

**Method:**

A curriculum development tool, the Mastery Rubric (MR), was created to structure the curriculum and career of the nurse practitioner (NP), the MR-NP. Cognitive task analysis (CTA) yielded the first of the three required elements for any MR: a list of knowledge, skills, and abilities (KSAs) to be established through the curriculum. The European guild structure and Bloom’s taxonomy of cognitive behaviors provided the second element of the MR, the specific developmental stages that are relevant for the curriculum. The Body of Work method of standard setting was used to create the third required element of the MR, performance level descriptors (PLDs) for each KSA at each of these stages. Although the CTA was informed by the competencies, it was still necessary to formally assess the alignment of competencies with the resulting KSAs; this was achieved via Degrees of Freedom Analysis (DoFA). Validity evidence was obtained from this Analysis and from the DoFA of the KSAs’ alignment with principles of andragogy, and with learning outcomes assessment criteria. These analyses are the first time the national competencies for the NP have been evaluated in this manner.

**Results:**

CTA of the 43 NP Competencies led to seven KSAs that support a developmental trajectory for instruction and documenting achievement towards independent performance on the competencies. The Competencies were objectively evaluable for the first time since their publication due to the psychometric validity attributes of the PLD-derived developmental trajectory. Three qualitatively distinct performance levels for the independent practitioner make the previously *implicit* developmental requirements of the competencies *explicit* for the first time.

**Discussion:**

The MR-NP provides the first articulated and observable developmental trajectory for the NP competencies, during and beyond the formal curriculum. A focus on psychometric validity was brought to bear on how learners would *demonstrate* their development, and ultimately their achievement, of the competencies. The MR-NP goes beyond the competencies with trajectories and PLDs that can engage both learner and instructor in this developmental process throughout the career.

## Introduction

Academic progression, readiness for clinical practice, consistent assessment criteria, student empowerment of learning with transparent expectations, curriculum design, lifelong learning goals, and career development are all purposes that would be served by an Advanced Practice Registered Nurse (APRN) developmental trajectory of the knowledge, skills and abilities (KSA) of an APRN practice from education preparation to expert clinician. Competency based education is a starting point to achieve this outcome. NP competency-based education has been at the forefront of the NP educators’ agenda since 1980, with the publication of *Guidelines for Family Nurse Practitioner Curricular Planning* and the subsequent development of the National Organization for Nurse Practitioner Faculty (NONPF). In 2014, NONPF published the *Nurse practitioner core competencies content*: *A delineation of suggested content specific to NP core competencies* [1]. This collection of specific competencies are grouped into nine content areas (scientific foundations (4); leadership (7); quality (5); practice inquiry (6); technology and information literacy (7); policy (6); health delivery system (7) ethics (3); and independent practice (13)).

Competencies are articulated for many health and health science programs, including advanced practice nursing [1], medical informatics and bioinformatics [2, 3], and medicine [4]. The National Organization of Nurse Practitioner Faculty (NONPF) and the American Association of Colleges of Nursing (AACN) have led the development of competencies for advanced practice registered nursing (APRN) education.

Competencies may be intermediate milestones [5] or endpoints for graduates of programs, but howsoever they are placed, they encourage “outcomes-based” education [6–10]. They generally capture what programs within a discipline must do, teach, and provide for graduates, bringing meaning and comparability across such programs [11]. Competencies have been articulated for the ‘main’ purpose of programs, for example in physical therapy [12], pharmacy [13], and bioinformatics [3].

As the articulation of competencies is increasing across disciplines, so is the recognition that competencies can be difficult to fully integrate into a curriculum [14]. The challenges have been highlighted by the disciplines of public health [15], medical education [16] and bioinformatics [3]. Graduate nursing education also experiences challenges related to developing competency-based education. The American Association of Colleges of Nursing (AACN) echoes the importance of competency-based APRN education while also reiterating the lack of a formal framework to develop competency-based education [17]. APRN education models have not significantly changed in over four decades. National APRN education leaders currently advocate the development of competency-based models of education; however, there is no current structure for consistent implementation of competency-based education [18–20]. Creating a framework to guide competency-based education is dauntingly complex.

The national discussion about competency-based education extends across clinical programs. In a June 2017 special issue of Medical Teacher, a set of 11 articles provide overviews of the wide variety of challenges faced by medical education as faculty worldwide seek to promote competency-based medical education (CBME). In that special issue, Holmboe et al. [21] specify that the organization of “teaching and learning experiences… to facilitate an explicitly defined progression of ability in stages” (p.575) is a fundamental characteristic of CBME. The same conclusion–that a list of competencies requires a supporting progression—was independently drawn for bioinformatics competencies as well [16]. The challenges associated with implementing competency-based education are not limited to medical [21] and bioinformatics [16] programs. An explicit and publicly shared developmental trajectory for learners *should* be a fundamental characteristic of any higher education curriculum. Ideally, this progression should be consistent with the principles of adult learning (andragogy), and decisions about who has, and who has not yet achieved a given stage, should be informed by psychometric considerations of validity.

A Mastery Rubric (MR) is a curriculum development and evaluation tool that specifically provides such a trajectory. The first MR was created in 2005 and published in 2010 [22] to describe the clinical research training curriculum, including application and admissions criteria, for a two-year certificate program. Every MR requires three elements: first is a list of knowledge, skills, and abilities (KSAs^1^) that the curriculum exists to impart, all of which are concretely observable and evaluable even though they can be both taught and demonstrated at the target level of performance in a variety of ways [23, 24]. A MR also requires an explicit developmental trajectory, within which performance levels (stages) are identified with sufficient concreteness that the third MR feature, performance-level descriptions (PLDs) of each KSA at each level or stage, can be articulated–and recognized when met. PLDs represent standards against which any individual student’s work can be evaluated. The creation of a MR integrates the KSAs with the developmental trajectory across stages that are programmatically meaningful, and that promote sustainable learning–i.e., learning that can continue after formal instruction ends and that can be transferred to new contexts [25].

A curriculum based on a MR is intended to support instruction and assessment that generate *actionable evidence* [26, 27]. *Actionable evidence* is information that can be used to make evidence-informed decisions. This would be beneficial to the institution for curriculum [28]; the instructor(s) about their course, a class session, or their assessments [29]; and students about their learning strategies [30, 31].

One of the most important features of a MR is that it is shared with all stakeholders, including the learners—so they can be engaged as full partners in their education. A MR-based curriculum can thereby engage the learner in metacognitive development, so that gaps or weaknesses in their performance or understanding, rather than feelings of satisfaction or unease, identify areas for more learning or practice. Instructors and learners can then use the MR to document that these gaps/weaknesses have been addressed [24, 26, 31]. A MR can support both the formal learning within a curriculum and also supporting the learners and other decision makers throughout the career [32]. Whether described for a career [32] or a curriculum [33], using a MR can support active learner engagement and explicit development towards the learning objectives of the curriculum. That is, instructors can use the MR to design/evaluate or revise a curriculum while the learner or graduate can continue to document their achievements of post-graduate levels of performance on all KSAs, as appropriate.

This paper describes a MR created for the curriculum and career of the nurse practitioner (NP). Intended to be shared with learners and all faculty, the MR-NP was designed to facilitate both curriculum design and ongoing assessment by *learners and* instructors, “to ensure that trainees continue to progress” [34] towards the achievement of the NP competencies, within the curriculum and beyond it, into the career.

## Methods

The MR-NP was developed in two phases, following formal methods. The first phase was a cognitive task analysis (CTA) to identify the constituent KSAs that underpin the core competencies articulated by the National Organization of Nurse Practitioner Faculties [1]. The CTA is explicated further in the (S1 Supplemental Materials). The second phase of developing the MR-NP was a facilitated standard-setting exercise with four expert nursing faculty, through which the PLDs were developed and refined. Both of these methods are described in greater detail below.

Once developed, validity evidence supporting the resulting MR-NP was then collected using Degrees of Freedom Analysis (DoFA) [35, 36]. The first DoFA assessed the alignment of the KSAs with the current NONPF competencies. The second DoFA validation explored the alignment of the MR-NP with the principles for learning outcomes articulation [27] for all participants (program, faculty, students). The final DoFA assessed alignment of a curriculum developed following the MR-NP with the principles of andragogy [37], and compared this alignment with that of a curriculum developed according to the original NONPF competencies *without* developmental considerations.

### Methods: Participants

The five co-authors were engaged in the development of the MR-NP from October 2015 to April 2017 as part of a Community of Practice focused on the stewardly scholarship of teaching and learning. The first author is the developer of the Mastery Rubric construct and a cognitive scientist specializing in higher and post graduate education with experience supporting the development of KSAs for Mastery Rubrics. The other four authors are experienced NP educators with post-graduate specialty certifications. In addition to maintaining active clinical practices they have been full-time educators in nursing across multiple institutional settings for at least five years (maximum 34 years). One co-author serves as Senior Scholar at our university’s center for teaching excellence and also serves in a leadership position for education innovation. Two co-authors are Program Directors of specialty tracks of the NP Master’s degree program. Together we served as the subject matter experts (SMEs) for the CTA.

### Methods: Cognitive task analysis

As noted, every MR requires a set of KSAs that the curriculum exists to transmit. An established method for understanding KSAs in any task or learning enterprise is a cognitive task analysis (CTA). There are five general steps in CTA: Collect preliminary knowledge/information; identify knowledge representations and organizations; elicit knowledge; analyze and verify data; and format results [38]. In the NP context, the first three steps of the CTA were already completed and published as the NONPF Competencies. These represent a rich dataset from which concepts, processes and principles were extracted (see S1 Supplemental Materials).

These first three CTA steps led to a first draft of KSAs based on the NONPF competencies, which used Bloom’s taxonomy to extract the minimum cognitive activities needed for each competency [40]. Analysis and verification of the KSAs was accomplished through face-to-face and online meetings to discuss the CTA results until consensus was reached. Specifically, we met bi-weekly to evaluate and work with the derived KSAs, using the NONPF competencies and the SMEs’ individual experiences as nurse practitioners and faculty. We also planned that, once the MR-NP was drafted (i.e., results formatted, CTA step 5), we would further verify these results by alignment (DoFA) of the final version of our KSAs with the NONPF competencies to ensure that every KSA was required for multiple competencies, and any KSA that did not support *at least two* competencies would then be evaluated for its relevance. The CTA was not going to yield KSAs that were directly representative of any competency, so assessing how the CTA results (i.e., the KSAs) aligned with those competencies was an important validation step for the KSAs.

### Methods: Standard setting

As described earlier, every MR requires a set of at least two developmental stages to be articulated for evaluable growth in each KSA, as well as performance level descriptors (PLDs) of each KSA at each of these stages. Together, the establishment of stages and the drafting of PLDs were accomplished through the standard setting exercise. The stages for the MR-NP were initially loosely based on decision-making steps in the NP program (admission to the program; admission to clinical preceptored experiences; graduation from the program; and two additional professional performance levels beyond graduation; see [24] for similar career-spanning stages in medical education).

The stages of most MRs [24] are modeled after the European guild structure: Novice, Apprentice, Journeyman [39]. This structure is chosen purposefully for MR staging because it captures a progressive sequence within which students can see themselves changing from less independent to more independent within the curriculum. Moreover, in this structure, the Apprentice is recognized as actively engaged in learning the craft or practice, while the Journeyman is the independent practitioner. These characteristics also signal to all instructors in a curriculum that students must *evolve* to progress–student evolution must be supported and the purpose of the curriculum is to facilitate these changes in observable and evaluable ways. Finally, recognizing that the end of a curriculum cannot represent the end of learning for the nurse practitioner, we supplemented the decision-making steps in the typical NP program with concrete career stages that represent distinct growth in responsibilities as well as abilities after graduation.

The final feature of a MR is drafting the PLDs. We iteratively articulated PLDs for each KSA across the decision points in the NP curriculum, and into the NP career, using a Body of Work approach [40]. Specifically, the first pass at drafting a PLD was "range finding" for performance of the KSA at a given stage, and the second pass was "pinpointing" [40], as described below. PLD drafting used Bloom’s taxonomy refined by appealing to the elements of psychometric validity in assessment, as outlined by Messick (1994) [41]:

1. What is/are the knowledge, skills, and abilities (KSAs) that students should possess at the end of the curriculum?
2. What actions/behaviors by the students will reveal these KSAs?
3. What tasks will elicit these specific actions or behaviors?

The integration of Bloom’s taxonomy and the Messick criteria in our standard-setting exercise facilitated the inclusion of concrete and observable behaviors in the PLDs that can be developed sequentially–and reinforced iteratively for deeper and sustainable learning over time. In our implementation of Messick questions 2 and 3, we considered what Bloom’s level the minimum specific actions/behaviors demonstrating that the KSA had been learned would need to be demonstrated for the learner to qualify for a given stage (e.g., “Apprentice”); this is the range-finding. The pin-pointing is accomplished by refining PLDs (see S1 Supplemental Materials) so that each KSA can be recognized at each stage for any sample of student work.

This approach ensures that a curriculum that is developed, or revised, to follow this developmental path for each KSA will likely support evaluable learning goals, and their achievement. Competencies, in general, are multi-dimensional and complex behaviors, whereas learning goals should be simple and not compound, among other things. This may be one reason why there has been difficulty in creating competencies-based educational experiences [3, 14–16]. The MR approach to curriculum development also increases the likelihood of *actionable* evidence [27, 28] for the institution as well as for instructors/course directors *and students*, because a MR is always intended to be made public and shared [36]. These features also add evaluability to the PLDs themselves, such that a curriculum or continuing education experience that follows the MR-NP (or one that promotes students following it) can be evaluated in terms of its achievement of intended results [6].

### Methods: Validation through Degrees of Freedom Analysis

Degrees of Freedom Analysis (DoFA) [35] is a qualitative method for assessing the alignment of data (observations) with predictions from theory or decisions [36, 42]. The recent modifications for using DoFA in educational contexts are specifically intended to summarize evidence surrounding decisions about teaching, learning, and assessment [36]. Validity evidence for the MR-NP was compiled from three DoFA matrices, assembling the evidence that it is:

1. sufficiently aligned with the NONPF competencies to conclude that a curriculum based on the MR-NP could support the achievement of these competencies;
2. consistent with principles of learning outcomes articulation and assessment, supporting a conclusion that a curriculum based on the MR-NP could promote actionable and evaluable learning outcomes, and
3. well-aligned with principles of andragogy, supporting a conclusion that a curriculum based on the MR-NP would also be so aligned.

Andragogic principles and *National Institute for Learning Outcomes Assessment* (NILOA) [27] characteristics are important, although underutilized, in higher, graduate, and professional education. The KSAs in the MR-NP, and their developmental trajectories, must support achievement of the consensus-based competencies if anyone would choose the MR-NP for curricular support. This validity evidence supports the decision to use the MR-NP in NP curriculum development.

## Results

The results of the Cognitive Task Analysis and standard setting exercise (i.e., the MR-NP) appear in Table 1. Table 2 presents the DoFA testing the alignment of the KSAs with the NONPF Competencies. Table 3 presents the DoFA alignment of the MR-NP with the NILOA criteria for actionable learning outcomes [27] while Table 4 outlines the alignment of the MR-NP with principles of andragogy [37] comparing this alignment with that of the competencies on which the MR-NP was based.

**Table 1:** Mastery Rubric for NP: Knowledge, skills, and abilities underpinning the nonpf competencies, and trajectories in their development.

LEVEL:	NP Novice (e.g., NP post-licensure program applicant)	NP Apprentice (e.g., prepared for precepted NP clinical work)	NP Journeyman-1 (e.g., recently licensed NP)	NP Journeyman-2 (e.g., Experienced NP)	NP Journeyman-3 (recognized NP leader)
LABELS:	Novice §	Apprentice	JOURNEYMAN		
General description of practitioner from each level	Has attained BSN level or licensed RN competencies. Appreciates the differences between NP’s and other nursing roles, but cannot identify what can/should be changed or improved from within the NP role. Experienced with meta-cognitive reflection in terms of their learning, their practice, and the potential for future professional development. Demonstrates skill to use patient care technology including information systems to support safe quality nursing practice. Understands the importance of continually improving the safety and quality of patient care at the BSN level.	Beginning to see the NP competencies that the curriculum targets as goals, and depends wholly on the curriculum to get them to those goals. Qualification for clinical work as an NP includes specific factual/scientific knowledge and abilities, and the individual is prepared to learn to synthesize this knowledge and abilities and utilize them appropriately in clinical practice (as an NP). Developing meta-cognitive reflection as an NP, in terms of their learning, their practice, and the potential for future professional development. Demonstrates a commitment to improving their skills to interact with information systems and technology, including accessing point of care resources for evidence-based NP decision-making to support patient care and safe NP practice. Understands the importance of continually improving the safety and quality of patient care at the NP level.	Meets NP licensing or certification requirements specific to the region/ jurisdiction. Possesses NP-specific factual/ scientific knowledge and abilities, and the individual has demonstrated the ability to synthesize these knowledge and abilities and utilize them appropriately in clinical practice. Demonstrates purposeful meta-cognitive reflection as an NP, in terms of their learning, their practice, and the potential for future professional development. Demonstrates competence with information systems and technology. Works to continually improve the safety and quality of patient care.	Maintains NP licensing or certification requirements and explicitly and purposefully incorporates the patient-specific context fully into decision making. Tailors practice to specific contexts, individuals, and situations based on clinical experience. Reliably identifies misspecification of methods chosen or employed. Chooses and executes correct diagnosis/management plan. Has a solid understanding of future professional development–what opportunities are needed and when they will be most advantageous for ongoing development and for improving their own practice. Demonstrates expertise with information systems and technology. Provides and directs safe care and improves the quality of patient care.	Exhibits the competencies of the NP curriculum at the highest level, including being able to identify multiple “other” experiences that are equivalent to what might be specified in the accreditation documentation. Reliably identifies misspecification of methods chosen or employed, and conceptualizes approaches to how to improve advanced nursing practice. Chooses and executes correct diagnosis/management plans, and flexibly responds to inconsistencies in patient or caregiver responses to these plans. Has an expert level understanding of future professional development–what opportunities are needed and when they will be most advantageous for ongoing development and for improving their own practice. Demonstrates and adaptively utilizes expertise with information systems and technology for the improvement and transformation of health care.
KSAS:					
**Prerequisite knowledge**	Demonstrate the ability to translate professional nursing science/knowledge into professional nursing practice—not yet at the NP level. Demonstrates competency with BSN-level ethical principles in practice and decisionmaking (where relevant), and written and oral work; inexperienced with the applications/ applicability of these principles in NP-level engagement. Beginning to operate consistently at Bloom’s levels 2–3 with respect to BSN knowledge and principles, including ethical, clinical decisionmaking.	Beginning to demonstrate the ability to translate professional nursing science knowledge and synthesize it with burgeoning NP science knowledge. Developing a sense of professional identity through familiarity with the new NP knowledge and its synthesis with existing knowledge. Operating at Bloom’s level 4 with respect to NS and NP core principles (patho, pharm, and advanced health assessment), including ethical, clinical decisionmaking. Demonstrates awareness of the relationships between NS and NP principles and the implications of these relationships for interprofessional teams and their evolving roles in them. Demonstrated engagement at Bloom’s level 4 with ethical principles in clinical decision-making and consequences of those decisions in the NP context.	Nursing Science plus NP science with minimal acceptable (demonstrated, and reflected upon) level of experience applying both together, synthetically if not smoothly and automatically. Beginning to operate at Bloom’s levels 5–6 with respect to NS and NP principles, including ethical, clinical decisionmaking. Prerequisite knowledge of these domains (plus specialties, if relevant) promote functioning as an ethical, licensed independent practitioner.	Nursing Science plus NP and advanced (# hours) level of experience applying both together and synthesizing them efficiently. Operating automatically, but reflectively, at Bloom’s levels 5–6 with respect to NS and NP principles; able to adjust these levels according to audience (patient, caregiver, colleagues). Prerequisite knowledge of these domains (plus specialties, if relevant) promote functioning as an ethical, licensed independent practitioner.	Automatic deployment of both Nursing Science and NP principles, fully synthesized with expert understanding of what can change/be changed about practice- and the ability to design, execute, and interpret an evaluable quality improvement study. Demonstrated ability to integrate new knowledge (in or outside of NS), into practice. Operating automatically, but reflectively, at Bloom’s levels 5–6 with respect to NS and NP principles; able to adjust these levels according to audience (patient, caregiver, community, colleagues, policy makers, and in scholarship). This performance represents functioning as an ethical, licensed independent practitioner.
Communication skills (including technology, literacies, and “humanities”)	English speaking and writing skills sufficient to clearly communicate with patients, caregivers/families and colleagues. Understands that “communication” is not just speaking and listening but also supports the development of sustainable therapeutic relationships with patients. Demonstrates basic interview skills and use of relevant medical terminology in professional nursing practice. Limited experience with motivational interviewing. Practice and experience with APA writing format. Competent reading and writing of/in scholarly work. Limited experience with scholarly presentation skills (written and verbal). Demonstrates skills to examine consumer health information for accuracy, timeliness, and appropriateness; and utilize relevant health information in patient education.	Able to communicate subjective and objective findings in patient documentation and orally to members of the health care team. Improving use of relevant medical terminology. Developing NP-level abilities to identify communication and cultural barriers and/or conflicts—for patients, families, colleagues, and within the system; recognizes challenges for themselves and their patients/families with respect to communication or cultural barriers and/or conflicts. Actively promotes the development of sustainable therapeutic relationships with patients and families. Introductory experience with motivational interviewing in NP practice. Developing their abilities to communicate across all scholarly work (written and verbal). Competence in using current APA format consistently. Understands health literacy concepts and is beginning to evolve skills to identify low health literacy. Developing awareness that low health literacy requires adjustment on the practitioner’s part. Translates technical, scientific, and lay health information so that it is appropriate for various users’ needs in the NP context.	Competent level of written and oral communication skills, including well-developed (i.e., through purposeful, reflective identification of new opportunities for growth) professional speaking and writing skills that always promote effective communication with patients, caregivers /families, and colleagues. Consistently develops sustainable therapeutic relationships with patients and across the healthcare continuum. Anticipates communication barriers and/or conflicts and is able to implement culturally & linguistically sensitive strategies for resolution. Competent use of relevant medical terminology. Proficient and independent with medical and motivational interviewing skills in NP practice. Competence in using current APA format consistently (correctly). Demonstrates basic scholarly presentation skills (written and verbal). Integrate health literacy strategies into their interactions with patients and families, including coaching for positive behavioral change. Understands the differences between information and health literacies, and can integrate appropriate technologies for knowledge management to improve health care.		Expert written and oral communication skills, including well-developed professional speaking and writing skills that always promote sustainable therapeutic relationships with patients, families, colleagues, and across the healthcare continuum. Anticipates potential communication barriers and/or conflicts and develops culturally & linguistically sensitive strategies to proactively address them. Expert use of relevant medical terminology. Experienced medical interviewing skills; Expert in motivational interviewing. Extensive experience with APA writing format; experience reviewing for journals or grant review panels. Scholarly presentation skills (written and verbal), with independent and collaborative scholarship. Evaluate health literacy strategies.
**Reflection and meta-cognition**	Reflection that is specific to BSN level nursing; developing awareness of their own existing metacognitive abilities. Initializing an appreciation that purposeful reflection and examination of their nursing practice is a metacognitive skill that is learnable, improvable, and important for their practice and competence.	Developing purposeful reflection skills specific to practice at the NP level. Engaged metacognition: consistently and actively seeking input and feedback–and new learning or practice opportunities- to address gaps in skills or reasoning. Developing evidence of actively seeking constructive feedback, and responding to it for both learning and practice.	Demonstrates metacognition and purposeful reflection, relating to learning, continuing professional development, and practice, specific to NP-level nursing. Consistently and actively seeking input and feedback from multiple perspectives–and new learning or practice opportunities- to address gaps in skills, practice needs, or reasoning. Demonstrates the highest level of accountability for professional practice. May take or create opportunities to start to develop their skills in fostering reflective practice in others.		Consistently and actively seeking input and feedback–and new learning or practice opportunities- to address gaps in skills or reasoning. Consistently engaged in self-directed learning and ongoing professional development. Demonstrates the highest level of accountability for professional practice.
**Data and Evidence skills**	Demonstrates understanding of basic research process. Able to evaluate credibility of limited information sources. With assistance, as a team member, able to participate in limited retrieval of appropriate data and evidence for a given case. Needs assistance with evidence analysis skills; functions as an informed consumer of research-and not as a producer. Limited statistical literacy. Can recognize a clinical nursing question at RN level for which data/evidence are needed. Able to contribute to a team that seeks to integrate data, evidence, and patient information to support patient management or quality improvement.	Demonstrates understanding of increasingly complex research questions and initiating abilities to address such questions. Developing both statistical literacy and the awareness that it is a learnable, improvable skillset. Minimal- competent- evidence analysis skills. Functions as a highly informed consumer of research, initializing development of the skills required to produce research. Ability to identify a question for which data and/or evidence are needed; ability to seek out data and evidence for a given case and ask for help if needed. Initializing the skills required to evaluate evidence-based literature for future clinical decision-making at the patient (not system or population) level.	Purposefully developing statistical literacy; acceptable evidence analysis and interpretation skills. Demonstrated ability to formulate research questions, seek out data and evidence for that question or a given case, and synthesize them into a coherent, defensible answer. Interpret evidence-based literature, discern validity and reliability of evidence, and integrate that evidence into clinical decision-making at the patient and family level. Able and willing to ask for specific help if needed, e.g., for additional diagnostic data, or assistance with statistics.		Statistically literate; competent evidence analysis and interpretation skills. Demonstrates skill in formulating relevant questions, and in seeking out data and evidence, as needed, for a given case and synthesize them to address their own questions as well as others, if asked. Always integrates data, evidence, and patient information into a coherent (correct) NP plan of care, or for quality improvement projects, or for systems or community improvement projects.
**Translation for evidence-based practice**	Integrates professional nursing science (ethics, biophysical, and psychosocial) into practice. May be able to conceptualize, with assistance, how to develop ideas for new practice approaches for professional nursing practice.	Developing awareness of advanced practice nursing science. Begins to describe current practice in terms of literature, evidence, data on practice and trends in healthcare. Begins to think critically about current practice approaches. Able to identify some of the data, evidence and patient information that are required for a coherent (correct) NP level plan of care—but not able to formulate that plan (independently).	Integrates advanced practice nursing science (ethics, biophysical and psychosocial) into practice. Able to identify and evaluate available literature, evidence and data on practice and epidemiology/trends in healthcare, in order to begin to develop ideas for new practice approaches. Able to independently identify and obtain, then integrate data, evidence, and patient information into a coherent (correct) NP plan of care. Able to determine if what seems like a new approach is actually new–or feasible—given a thorough evaluation of specific, relevant research, theory, and practice. Developing and refining the ability to justify choices of apparently comparable approaches in care.		Expertly integrates advanced practice nursing science (ethics, biophysical, and psychosocial), as well as organizational science, together with all relevant evidence and practice from other specialties into their practice. Evaluates all available literature, evidence, and data on practice and epidemiology/ trends in healthcare, and both A) identifies data or evidence that are lacking to support a decision (and then seeks that data/evidence); and B) develops new practice approaches based on integration of research, theory, and practice; also develops evaluable tests of these new practice approaches.
**Clinical practice**	Effective practice at professional nurse level. Able to justify choices of apparently comparable approaches in professional nursing. Demonstrates professional behavior appropriate for practice setting.	Application of pre-req knowledge, communication, reflection and metacognition and translation to practice KSAs and able to use independent judgment (including determining that they need assistance). Demonstrates professional APN behavior and begins to assume APN role in clinical practice. Conducts basic and systematic assessments of undifferentiated patients, inclusive of health promotion, disease prevention, with emerging consideration of contextual issues (ethics, family dynamics, socioeconomic, culture). For common health problems begin initiation of plan of care by formulating a problem list and differential diagnoses, initiate a plan of care with pharmacologic and nonpharmacologic interventions. Growing therapeutic communication skills, including interviewing, patient education, motivational interviewing, in NP practice.	Conducts comprehensive and systematic evaluation of complex patients with common diagnoses, inclusive of health promotion, disease prevention, and acute or chronic management, with consideration of contextual issues (ethics, family dynamics, socioeconomic, culture); design, implement, and evaluate individualized evidence- based interventions based on this assessment. Provides patient care that improves healthcare delivery and outcomes, including access, quality and cost. Participates in change initiatives that improve patient outcomes. Collaborates with multiple stakeholders (patients, families, integrated health care teams) to improve health outcomes across the continuum of care.	Autonomously conducts comprehensive and systematic evaluation of complex patients, inclusive of health promotion, disease prevention, and acute or chronic management, with consideration of all contextual issues (ethics, family dynamics, socioeconomic, culture); design, implement, and evaluate individualized evidence based interventions based on this assessment. Leads patient care and change initiatives that improve health care delivery and outcomes, including access, quality and cost. Assumes leadership roles to foster collaboration with multiple stakeholders (patients, families, integrated health care teams) to ensure continuity of care for the patient and family to improve health outcomes.	Expertly, automatically, and autonomously conducts comprehensive and systematic evaluation of complex patients, with consideration of all contextual issues (ethics, family dynamics, socioeconomic, culture); design, implement, and evaluate individualized evidence based interventions based on this assessment. Functions as a consultant to facilitate and improve NP practice. Incorporates principles of business, finance, and economics while expertly and automatically providing patient care and leading system changes that improve healthcare access, delivery, quality and outcomes. Assumes complex and advanced leadership roles to foster collaboration with multiple stakeholders (patients, communities, integrated health care teams and policy makers) to guide and initiate change to ensure continuity of care across the healthcare continuum. Improves population health outcomes at the systems level by assessing health systems, designing solutions and evaluating outcomes.
**Policy/** **Advocacy**	Understanding BSN scope of practice and the policies that shape it. Recognize that policy and advocacy are important to guide practice. Recognize unit and institutional policy needs/gaps at the professional nurse level.	Beginning understanding of policy and its role in NP practice. Identifies professional organizations and activities that influence advanced practice nursing and/or health outcomes of population focus. Recognize the intended aim of existing health policies related to NP practice. Recognize a professional policy need/gap and able to explore existing policies and initiate conversations about solutions. Articulates a need or gap but not able to initiate change.	Describe the rationales and impacts of public policy on the health of well-being of patients and families. Participates in professional organizations and activities that influence advanced practice nursing and/or health outcomes of population focus. Critiques existing health policies at all levels. Developing advocacy skills to address the policy need/gap through partnerships with stakeholders related to policy development, evaluation and leadership to improve healthcare delivery and outcomes including access, quality and cost.		Leads professional organizations and activities that influence advanced practice nursing and/or health outcomes of population focus. Develop, implement and critically analyze health policies at all levels (institutional, local, state, federal, and/or international) from perspective of multiple stakeholders Demonstrate advocacy skills related to policy development, evaluation and leadership to improve healthcare delivery and outcomes, including access, quality and cost.

Notes:

^§^ Characteristics of the “novice” for all KSAs are consistent with the BSN essentials (American Association of Colleges of Nursing, 2008 [45].

**Table 2:** Alignment of MR-NP KSAs with the NONPF competencies whose development a MR-NP based curriculum will support.

MR-NP KSA: NONPF Competency:	PRE-REQUISITE KNOWLEDGE *	COMMUNICATION	REFLECTION & METACOGNITION	DATA & EVIDENCE	TRANSLATION FOR EBP	CLINICAL PRACTICE	POLICY/ ADVOCACY
**Scientific foundations**							
Critically analyzes data and evidence for improving advanced nursing practice.				√	√	√	
Integrates knowledge from the humanities and sciences within the context of nursing science.	√	√	√	√	√		
Translates research and other forms of knowledge to improve practice processes and outcomes.	√	√	√	√	√	√	√
Develops new practice approaches based on the integration of research, theory, and practice knowledge.		√	√	√	√	√	√
**Leadership**							
Assumes complex and advanced leadership roles to initiate and guide change.	Competency is not concrete enough to identify specific KSAs that support its achievement.						
Provides leadership to foster collaboration with multiple stakeholders (e.g. patients, community, integrated health care teams, and policy makers) to improve health care.		√				√	
Demonstrates leadership that uses critical and reflective thinking.		√	√	√		√	√
Advocates for improved access, quality and cost effective health care.		√		√		√	√
Advances practice through the development and implementation of innovations incorporating principles of change.		√		√	√	√	√
Communicates practice knowledge effectively, both orally and in writing.	√	√				√	
Participates in professional organizations and activities that influence advanced practice nursing and/or health outcomes of a population focus.							√
**Quality Competencies**							
Uses best available evidence to continuously improve quality of clinical practice.		√		**√**	√	**√**	√
Evaluates the relationships among access, cost, quality, and safety and their influence on health care.	√	√					
Evaluates how organizational structure, care processes, financing, marketing, and policy decisions impact the quality of health care.	√	√				√	√
Applies skills in peer review to promote a culture of excellence.		√	√			√	√
Anticipates variations in practice and is proactive in implementing interventions to ensure quality.			√	√	√	√	
**Practice Inquiry Competencies**							
Provides leadership in the translation of new knowledge into practice.	√	√	√	√	√	√	√
Generates knowledge from clinical practice to improve practice and patient outcomes.	√	√	√	√	√	√	√
Applies clinical investigative skills to improve health outcomes.	Competency is not concrete enough to identify specific KSAs that support its achievement.						
Leads practice inquiry, individually or in partnership with others.		√	√	√		√	
Disseminates evidence from inquiry to diverse audiences using multiple modalities.		√		√			
Analyzes clinical guidelines for individualized application into practice.				√		√	
**Technology and Information Literacy Competencies**							
Integrates appropriate technologies for knowledge management to improve health care	√						
Translates technical and scientific health information appropriate for various users’ needs.	√	√					
Assesses the patient’s and caregiver’s educational needs to provide effective, personalized health care.		√	√			√	
Coaches the patient and caregiver for positive behavioral change.	√	√				√	
Demonstrates information literacy skills in complex decision making.	√		√	√			
Contributes to the design of clinical information systems that promote safe, quality and cost effective care.	Competency is not concrete enough to identify specific KSAs that support its achievement.						
Uses technology systems that capture data on variables for the evaluation of nursing care.	Competency is not concrete enough to identify specific KSAs that support its achievement.						
**Policy**							
Demonstrates an understanding of the interdependence of policy and practice.	√						√
Advocates for ethical policies that promote access, equity, quality, and cost.	√	√		√			√
Analyzes ethical, legal, and social factors influencing policy development.		√		√			√
Contributes in the development of health policy. *	√*						√
Analyzes the implications of health policy across disciplines.							
Evaluates the impact of globalization on health care policy development.	√	√	√	√			√
**Health Delivery System**							
Applies knowledge of organizational practices and complex systems to improve health care delivery.	√			√		√	
Effects health care change using broad based skills including negotiating, consensus-building, and partnering.	√	√	√	√	√	√	
Minimizes risk to patients and providers at the individual and systems levels.	√	√		√	√	√	
Facilitates the development of health care systems that address the needs of culturally diverse populations, providers, and other stakeholders.	Competency is not concrete enough to identify specific KSAs that support its achievement.						
Evaluates the impact of health care delivery on patients, providers, other stakeholders, and the environment.	√			√			
Analyzes organizational structure, functions and resources to improve the delivery of care.	√			√	v	√	
Collaborates in planning for transitions across the continuum of care.	√	√			√	√	
**Ethics**							
Integrates ethical principles in decision making.	√		√	√		√	
Evaluates the ethical consequences of decisions.	√		√	√		√	
Applies ethically sound solutions to complex issues related to individuals, populations and systems of care.	Competency is not concrete enough to identify specific KSAs that support its achievement.						
**Independent Practice**							
Functions as a licensed independent practitioner.		√	√	√	√	√	√
Demonstrates the highest level of accountability for professional practice.			√	√	√	√	
Practices independently managing previously diagnosed and undiagnosed patients.		√	√	√		√	
Provides the full spectrum of health care services to include health promotion, disease prevention, health protection, anticipatory guidance, counseling, disease management, palliative, and end-of-life care.		√	√	√	√	√	
Uses advanced health assessment skills to differentiate between normal, variations of normal and abnormal findings.		√	√	√		√	
Employs screening and diagnostic strategies that are in line with evidence-based practices in the development of diagnoses.			√	√	√	√	
Prescribes medications within scope of practice.		√	√	√	√	√	
Manages the health/illness status of patients and families over time.		√		√		√	
Provides patient-centered care recognizing cultural diversity and the patient or designee as a full partner in decision- making.	√	√	√	√		√	
Works to establish a relationship with the patient characterized by mutual respect, empathy, and collaboration.		√	√			√	
Creates a climate of patient- centered care to include confidentiality, privacy, comfort, emotional support, mutual trust, and respect.		√	√			√	
Incorporates the patient’s cultural and spiritual preferences, values, and beliefs into health care.	√	√	√			√	
Preserves the patient’s control over decision making by negotiating a mutually acceptable plan of care.		√	√	√		√	√

Notes:

* pre-requisite knowledge is indicated as a key KSA underpinning successful achievement of a competency in this table if there is specialized knowledge *beyond what the typical curriculum can be expected to convey* that must be utilized. For example, pre-requisite knowledge is identified for the penultimate competency, “incorporates the patient’s cultural and spiritual preferences, values, and beliefs into health care” because the patient’s cultural and spiritual preferences, values and beliefs may be quite far outside the candidate’s experience and these may be unlikely to have been taught in any given NP program.

**Table 3:** Alignment of Principles for documenting and improving assessment (NILOA, 2016) [27] with features of the MR-NP from student and institutional or programmatic perspectives.

	How does the MR-NP follow the NILOA criteria?	
PERSPECTIVE: Principles for Learning Outcomes generating actionable evidence:	STUDENT PERFORMANCE	PROGRAMMATIC EFFECTIVENESS
Develop/articulate specific actionable learning outcomes	MR-NP helps students identify their progress towards articulated learning objectives at every point in the curriculum. The competencies do not provide this reference.	MR-NP helps instructors and institutions identify and articulate developmental learning objectives; the competencies do not include developmental trajectories. The alignment of these objectives with current–*and future*—competencies is visible and actionable with the MR-NP.
Connect learning goals with student work	If work or performance is not concretely aligned with the curriculum learning goals in the MR-NP, students see this and can remediate that (with additional work or training). With the competencies alone, learners cannot determine if they are “on track” to achievement.	If learning goals are not reflected in student work (assignments), instructors/institution can use the MR-NP to see this and remediate with different assignments. Without the MR-NP, milestones and decisions are based solely on yes/no determinants of whether the learner achieved a given competency.
Articulate learning outcomes collaboratively	Students see in the MR-NP what the curriculum is designed to do, and if they perceive they are not achieving the stated learning outcomes, they can act to achieve them on their own initiative. With competencies, students only see where they need to end up.	With both the MR-NP and competencies, faculty across the curriculum see what are its intended outcomes. The MR-NP facilitates instructors in courses that follow a sequence collaborating to ensure that earlier student work prepares students for later assignments. The MR-NP, but not the competencies alone, enable the entire institution to support the achievement of the NONPF competencies.
Outcomes support assessment that generates actionable evidence	With the MR-NP, students can/are encouraged to actively self-assess, to ensure they are making progress on the developmental path. With no path, the competencies alone cannot engage students.	Institutions and instructors see explicit alignment of curricular features (courses, assignments/work products) and can use this evidence to support or change the approach using the MR-NP. This is not facilitated by the competencies.
Outcomes are focused on *improvement*	The explicit articulation of expected growth and development in the target KSAs that the MR-NP produces focuses all stakeholders on improvement in student performance of these KSAs–emphasizing they are not static. This is not possible with the competencies alone.	
Outcomes document learning and its extent	Learners generate evidence of their achievement and ongoing development of KSAs using the MR-NP. Learners cannot do this with just the competencies.	Both the MR-NP and the competencies allow instructors and institutions to structure training/teaching to generate documentation of learning and the achievement of articulated learning objectives. The developmental features of the MR-NP facilitate instructional support of that development.
Outcomes provide evidence of quality of learning	A portfolio can be created articulating the extent and quality of learning; with the MR-NP the portfolio can be formative and focus on development; with just the competencies the portfolio functions summatively only.	Assessment opportunities that document the achievement and quality of learning can be developed using either the MR-NP or the competencies, but the competencies offer only summative opportunities for assessment while the MR-NP supports formative assessment as well.
Expectations are explicit in the outcomes	The MR-NP makes explicit the expectation that the learner takes some responsibility for self-assessment and ensuring ongoing development until the target performance level is achieved.	The MR- NP makes explicit the institutional obligation to provide learning opportunities that can and do promote growth and development in the target KSAs.
Evidence from the outcomes is externally relevant	Portfolios documenting the achievement of learning outcomes throughout the curriculum, and/or at specific milestone “moments” (e.g., when determining readiness to enter precepted clinical training) can be used to document readiness/qualification to proceed.	Integrating competencies into curricula is known to be challenging. Institutions that adopt the MR- NP and use it to guide curriculum development or evaluation can document their alignment of learning outcomes with the current (2016) competencies, and can easily plan for *evaluable* changes when competencies change in the future.

Table adapted from Tractenberg, 2017-a [26] with permission.

**Table 4:** Comparison of how the principles of andragogy are aligned with, or met by, a curriculum based on the NONPF competencies vs on the MR-NP.

Principle:	How met in a curriculum based on Competencies	How met in a curriculum based on MR-NP
*Adults are self-directed and internally motivated*, *and so can—and need to—take responsibility for choices that further learning objectives*.	Competencies can be shared with learners, and provide “endpoints”; once achieved, competencies can be “checked off”. No guidance on how learners can be shaped throughout a curriculum.	The MR-NP with its developmental trajectory is shared. Curriculum is designed to promote learner comprehension of why material and reasoning is important as well as to engage the learner in actively building towards successive performance levels.
*Adults bring prior knowledge and experience to learning–and seek to connect new information with prior learning*.	Has potential to promote the seeking of (new) opportunities to demonstrate competencies.	Designed to promote autonomous engagement and self-directed progression through the developmental trajectory on each KSA.
*Adults are goal oriented*, *and require explicit*, *recognizable*, *and achievable learning goals*.	The competencies are, or may be inferred to be, endpoints.	Each stage in the MR-NP explicitly builds on prior experience. Because the entire trajectory is articulated, the learner can develop a mental model of the target level of performance they desire.
*Adult learners need to know* *why* *they are learning what is presented*.	If perceived to be endpoints, competencies may not actively promote an attention to ongoing skills-building.	The developmental trajectory in each KSA promotes self-assessment of the readiness to learn, as well as the recognition that the KSAs can grow at different rates–and must be integrated in order for competencies to be achieved.
*Adult learners benefit from practical*, *authentic assessments and practice experience*.	Competencies are highly applied, problem-centered, and contextual. They can support empirically- and theoretically- optimized learning opportunities.	KSAs and the developmental trajectories for each are not contextual *by design*, so multiple contexts can be used to deepen and demonstrate the KSAs over time.
*Adults are motivated to learn but need to be treated as partners in the learning enterprise*, *not as vessels to be filled*.	The competencies have important intrinsic value as consensus-derived indicators of professional achievement. However, when competencies are treated as items to be checked off a list, the motivation may tend towards checking these off, rather than towards initiating ongoing learning and development.	The MR-NP is constructed to promote personal and individual engagement, by faculty as well as students, in each student’s achievement of the competencies of advanced practice nursing. This engagement may be more challenging than current curricula, so the initial perception of intrinsic value may be difficult to perceive.

### Results: Cognitive task analysis

The cognitive task analysis (see S1 Supplemental Materials) initially identified five KSAs as underpinning the NONPF competencies: prerequisite knowledge; communication skills; reflection and metacognition; data and evidence skills; and clinical practice. Two additional KSAs were identified and included/retained: One was needed to accommodate NONPF competencies that fell between “data and evidence skills” and “clinical practice”: this became “translation for evidence-based practice”. The other was needed to capture engagement with policy and advocacy:“policy/advocacy.” As the developmental paths through the performance-level descriptors for these KSAs were developed, both of them retained their differentiations from the other KSAs. All seven KSAs are presented in the MR-NP shown in Table 1.

### Results: Standard setting

The Body of Work approach [40] to standard setting, i.e., writing the PLDs that capture standards of performance on each KSA at each stage, was used to draft and revise descriptions of how a “minimally competent individual” [43] would carry out the KSAs at each stage to demonstrate that they were capable of performing at that level.

As can be seen in Table 1, the MR-NP represents the stages in the developmental trajectory of the NP from admission to the program (“Novice”) through their qualification for clinical precepted experiences (“Apprentice”), graduation (“Journeyman 1”) and across their career trajectory (“Journeyman 1” through “Journeyman 3”). Development is implicit, but not articulated, in the NONPF Competencies.

The NP curriculum decision points were used in the first drafts of PLDs, and the final PLDs were revised specifically to characterize the individual at these stages: accepted into a formal training program (“Novice”), the individual who can document their preparation for preceptored clinical experiences (“Apprentice”) and the individual who is newly independent (“Journeyman”). Evidence for admission into an NP program in the United States (based on the requirements for Bachelor’s of Science for Nursing or BSN [44]), and preparedness for clinical preceptorship are consistent with novice and apprentice level performance respectively; graduation from the NP program can be considered to represent an individual’s qualification for independent practice. The complex nature of professional development for the NP beyond the end of formal education is recognized by the inclusion of three distinct levels of “Journeyman”; these were dictated in part by the opportunities and requirements that the NONPF competencies themselves suggest.

The first row of the MR-NP provides a general description of overall performance of the minimally-qualified individual at each level [45]. Once the KSAs were extracted and PLDs were drafted for the Novice level, we determined that the PLDs were consistent with our program’s admission process, providing content validity for that set of PLDs. Three distinct Journeyman stages emerged: the recent graduate is described by Journeyman-1, the individual who has NP *experience* that must accrue after graduation is at the Journeyman-2 level, and Journeyman-3 describes the recognized NP leader with NP practice *expertise*. All Journeyman-level individuals are qualified for independent practice; the fact that the standard setting led to three differentiable levels of independence is a function of the competencies themselves. As can be seen in Table 1, the PLDs of all KSAs except “prerequisite knowledge” and “clinical practice” are the same for Journeyman 1–2. Journeyman 3 performance level descriptors characterize the practitioner with sufficient experience to be considered a “recognized leader,” qualitatively different from Journeyman 1 and 2. These levels of performance differ as experience changes to expertise–making *implicit* developmental requirements of the NONPF competencies, beyond the formal curriculum and throughout a career, *explicit* for the first time.

### Results: Validity evidence from DoFA

Validity evidence for the MR-NP was obtained by appeal to extant criteria. First, Table 2 explores alignment of the KSAs with the individual NONPF competencies.

As Table 2 reflects, most of the 49 competencies articulated in the NONPF statement [1] are supported by at least one KSA. However, not all of these competencies are specifically dependent on the KSAs outlined in the MR-NP. We found that six of 49 competencies could not be aligned with any of the KSAs in the MR-NP; the remaining 87% of the NONPF competencies were aligned with at least two KSAs. Failures in alignment between the KSAs and the competencies were identified independently by different co-authors. Two characteristics were determined to have prevented alignment of KSAs with these six competencies. Either the competency was determined not to have been described concretely enough to identify a clear KSA or instructional approach, or else the competency was evaluable, and did not require any of the KSAs in order to be achieved. These are identified as such within Table 2.

Further validation of the MR-NP was sought by appeal to the 2016 recommendations for actionable learning outcomes published by the National Institute for Learning Outcomes Assessment (NILOA, [27]). NILOA was established to encourage educators nationwide to develop evaluable and robust learning outcomes to improve student learning and higher education generally. Their 2016 policy statement [27] includes five important attributes of meaningful learning outcomes. Table 3 presents the alignment of the MR-NP features with the predictions derived from these five NILOA principles for documenting learning outcomes, plus four additional characteristics of robust learning outcomes highlighted in Tractenberg, et al. (2017) [25].

Table 3 shows how the MR-NP can be used to ensure that learning outcomes for courses and the curriculum are as explicit for students as they are for faculty and the program or institution. A curriculum that is created or revised using the MR-NP will exhibit all five of the NILOA learning outcome characteristics, generating actionable evidence for students as well as for the program and faculty engaged in the curriculum. However, a curriculum that is based on just the NONPF competencies will *not* generate such evidence. This is a function of the implicit nature of any developmental trajectory in the competencies; the MR-NP makes development *explicit*, and the PLDs were formulated specifically to conform to psychometric validity [34, 41]. The competencies offer only summative opportunities for assessment while the MR-NP supports formative assessment as well.

Finally, a MR is intended to promote curriculum development, evaluation, or revision that is consistent with principles of andragogy [37]. Table 4 is a descriptive analysis that presents how the MR-NP embodies this alignment.

Table 4 shows concrete differences in alignment with andragogical principles that would arise from a curriculum that is developed to feature the NONPF competencies [1] and one that was developed (or revised) using the MR-NP. This is not an empirical analysis, but a conceptual one. While *both* types of curricula could lead students to achieve the NONPF competencies, the curriculum developed using the MR-NP would also be consistent with the principles of andragogy, thereby engaging learners as partners in their own development and promoting reflection and metacognition to a greater extent [25].

## Discussion

A new MR was created using published national competencies for nurse practitioner (NP) education, together with a formal and iterative standard setting exercise. Seven key areas of knowledge, skills, and abilities (KSAs) were derived so that an NP curriculum can go *beyond* simply aligning with, or including, the competencies. The MR-NP (Table 1) can be used to develop and evaluate courses and curricula, as well as teaching, learning, and practice. It provides a roadmap to addressing many challenges in NP education: supporting the explicit, but flexible, articulation of valid learning outcomes; promoting growth in students along a newly-specified developmental trajectory; and leading to the achievement of the target competencies—in the curriculum and beyond, into the career of the NP. The structure can be used to create consistency across programs, faculty, and students, enabling specificity and measurability in assessment. By making expectations transparent to all stakeholders, the MR-NP empowers self-directed growth.

The MR-NP has face validity that derives partly from its support of/alignment with the national competencies that are at its core (Table 2), with additional validity accruing from the application of cognitive task analysis emphasizing Bloom’s taxonomy [46] with formal and iterative standard-setting. The infusion of psychometric validity in the PLDs yields a usable *and evaluable* curriculum description (Table 1). The DoFA analysis on the consistency of the MR-NP, with principles of learning outcomes assessment (Table 3), highlights how existing NP curricula can be revised to generate evidence about learning for both the student *and* the instructor/institution; these revisions are not clear with the competencies but are accessible with the MR-NP. This DoFA replicates the results from two other MRs for Statistical Literacy [26] and for Ethical Reasoning [32], suggesting that a curriculum that employs the MR approach can promote this actionable evidence. Finally, a curriculum that utilizes the MR-NP is aligned with principles of andragogy (Table 4) in ways that a curriculum that utilizes just the competencies would not be, even if the competencies are shared with learners as fully as the MR-NP is intended.

The creation of the MR-NP is the first step in our educational research program, and as such, we have not had the opportunity to empirically study curricula that do and do not utilize it. The methodology and formal incorporation of psychometric validity, learning outcomes assessment, andragogy principles, and cognitive complexity will hopefully encourage NP curriculum developers to adopt the MR-NP and report on their results.

A MR can engage the learners in the structure and achievement of learning objectives *throughout their career* [32]. While the competencies represent targeted end states, the MR-NP promotes ongoing engagement with its core KSAs past graduation and well into NP professional practice and ultimately, leadership roles. By developing concrete, measurable objectives that are supportive of the NONPF competencies—across an explicit educational trajectory—nurse faculty and students can document individual accomplishment of competencies as they are developed over time. Students who are not achieving competencies as expected can/may identify the need and opportunity for remedial work focused on the area of deficiency rather than repeating an entire course or an additional prescribed number of clinical hours.

Faculty development and advancement, curriculum evaluation, and classroom innovation can each/all be facilitated when *curricular structure* is used to align and sequence courses and learning objectives. When formal structure explicitly underpins all components of a curriculum like the MR-NP suggests, both teaching and learning can be made more evaluable. This evaluability can strengthen the curriculum generally, while simultaneously augmenting the likelihood of the success of all students. Anecdotally, all of these claims were supported when the practitioner co-authors utilized the MR-NP in their teaching, mentoring, and student assessment. Because these experiences were not formal or systematic, they are offered as preliminary evidence that encourage future formal studies regarding the implementation and usability of the MR-NP.

The MR-NP flexibly represents how the NONPF competencies, including the most cognitively-complex ones, can be achieved within the NP curriculum by integrating a developmental trajectory that increases the cognitive complexity of instruction and work products across the curriculum and NP career. This MR-NP provides a straightforward, useful tool for instructors to use when revising their objectives and syllabi. The usability of the MR-NP increases the potential for buy-in and uptake within APRN programs. Buy-in, commitment of time and resources, and a formal evaluation plan are all required if the integration of competencies is to be done for all courses in the curriculum [47].

The MR-NP provides clear descriptors of when an APRN student is competent to transition into supervised clinical practice and provided a framework for demonstrating that competencies for independent clinical practice have been met. Without a developmental trajectory, the competencies alone might not support the “transition to practice” [48]; however, the trajectory outlined by the MR-NP might. Our future work will focus on examining the uptake and buy-in to this particular MR-NP at our institution beyond our research group, and the generation of actionable evidence that supports the accreditation claim that the target NONPF competencies are not simply aspirational, but *descriptive* [49]. The support of the MR-NP of transition to practice is another avenue for research.

In conclusion, these results support the use of an explicit developmental trajectory, like the MR-NP, to promote learning and assessment to ensure that competencies are both achieved by students *and* explicitly supported throughout the curriculum and throughout the transition to practice.

## Supporting information

S1 Supplemental Materials (DOCX)
