# Supplementary material for: A developmental trajectory supporting the evaluation and achievement of competencies: Articulating the Mastery Rubric for the nurse practitioner (MR-NP) program curriculum
Source: PLoS One. 2019 Nov 7;14(11):e0224593. doi: 10.1371/journal.pone.0224593 (PMC6837290; doi:10.1371/journal.pone.0224593)
Supplement: S1 Supplemental Materials — (DOCX) [file pone.0224593.s001.docx]

**Supplemental Materials**. Cognitive Task Analysis Methodology

There are five general steps in cognitive task analysis (CTA, discussed in Clark et al. 2008; p.580). The table below lists these steps and describes the contributions to each step from the subject matter experts (co-authors, one cognitive scientist and four expert practitioners and educators), the relevant theory, and from the literature. The following figure outlines the method by which the CTA led to KSAs.

| CTA steps^1^: | Subject Matter Experts (SMEs) | Theoretical contributions | From the literature |
| --- | --- | --- | --- |
| 1.Collect preliminary knowledge/information | evaluation of Blooms-level requirements in each element (RET); SMEs (AWB, MRW, TPP, JBR) parsing undergraduate [44] and NP competencies [1]; | Bloom et al. [46]  Messick [41] | Standard setting models and methods [43] |
| 2. Identify knowledge representations and organizations | Guild structure; career stage (stages of degrees) plus existing NP curriculum; | Bloom’s [46] Messick [41] | NONPF Competencies [1]; Body of Work standard setting [40] |
| 3. Elicit knowledge | Performance level descriptor drafting/revising via standard setting | Bloom’s [46] Messick [41] | Accreditation, curriculum, & training/workforce development considerations across educational contexts |
| 4. Analyze and verify data | SMEs engage in iterative range-finding, pinpointing, alignment of KSAs, stages, and other PLDs; validation with alignment of KSAs and guidelines. Iterative and ongoing analysis and revisions of PLDs by all co-authors for observability and relevance. | | |
| 5. Format results | Following format for MR construct; SME iteration on PLDs to insure clarity and relevance without redundancy. | | |
| ^1^Adapted from Clark et al. 2008 [38]; p.580 | | | |

Figure. Iterative process of applying CTA to NONPF competencies [1] derive KSAs for the MR-NP.


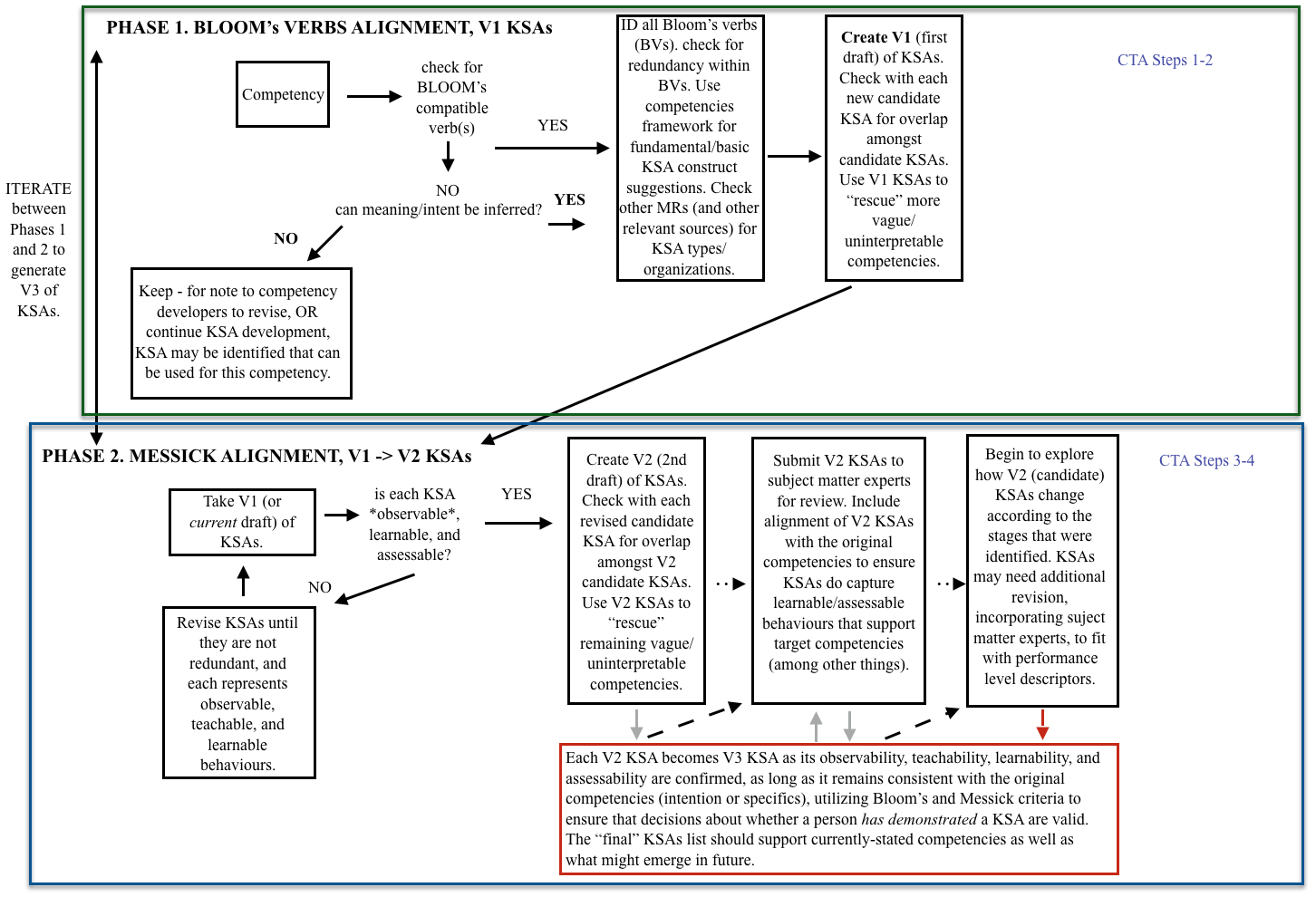


1 Thomas, A., Crabtree, M. K., Delaney, K., Dumas, M. A., Kleinpell, R., Marfell, J, et al. *Nurse practitioner core competencies content: A delineation of suggested content specific to NP core competencies*. 2014. http://c.ymcdn.com/sites/nonpf.site-ym.com/resource/resmgr/Competencies/NPCoreCompsContentFinalNov20.pdf

38 Clark RE, Feldon DF, van Merriënboer J, Yates KA, Early S., *Cognitive Task Analysis* in J. Michael Spector, M. David Merrill, Jan Elen, M. J. Bishop Eds. *Handbook of research on educational communications and technology*, 3^rd^ ed. 2008. Mahwah, NH: Lawrence Earlbaum Associates. 577-593.

41 Messick, S., *The Interplay of Evidence and Consequences in the Validation of Performance Assessments.* Educational Researcher, 1994. **23**(2): p. 13-23.

43 Plake BS, Cizek GJ. *Variations on a Theme: The modified Angoff, Extended Angoff, and Yes/No standard setting methods.*, in *Setting performance standards. Foundations, methods, and innovations*, GJ Cizek, Editor. 2012. New York, NY: Routledge. Pp 181-199.

44 American Association of Colleges of Nursing, *The Essentials of Baccalaureate Education for Professional Nursing Practice*. 2008.

46 BS Bloom (Ed.), with Engelhart MD, Furst EJ, Hill WH & Krathwohl DR. *Taxonomy of educational objectives: Handbook I: Cognitive domain.* 1956. New York, NY: David McKay.
